# Supplementary material for: Stem Cell Intrinsic Hexosamine Metabolism Regulates Intestinal Adaptation to Nutrient Content
Source: Dev Cell. 2018 Oct 8;47(1):112–121.e3. doi: 10.1016/j.devcel.2018.08.011 (PMC6179903; doi:10.1016/j.devcel.2018.08.011)
Supplement: Document S1. Figures S1–S4 and Table S3 [file mmc1.pdf]

Developmental Cell, Volume *47*

Supplemental Information

Stem Cell Intrinsic Hexosamine Metabolism Regulates Intestinal Adaptation to Nutrient Content

Jaakko Mattila, Krista Kokki, Ville Hietakangas, and Michael Boutros

FIGURE S1 related to figure 2

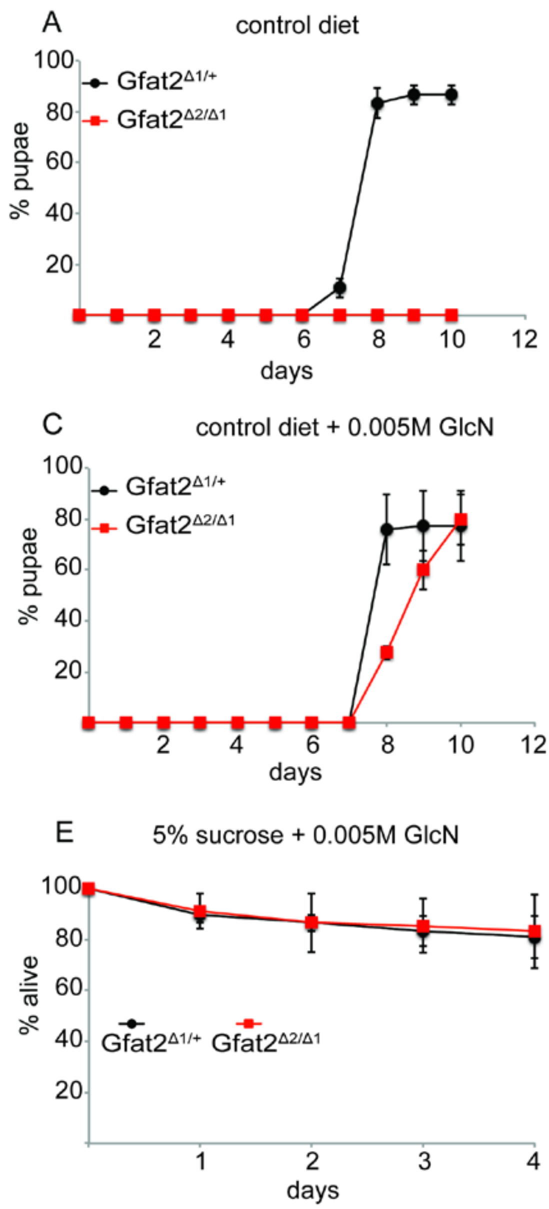

Figure S1. ***gfat2*** **mutant lethality is rescued by GlcNAc feeding. Related to Figure 2.**

(A-C) Pupation curves of *gfat2*$^{\Delta1/\Delta2}$ trans-heterozygote and *gfat2*$^{\Delta1/+}$ controls fed in control (A) and GlcNAc supplemented diets (B & C). (D-E) Lethality of *gfat2*$^{\Delta1/\Delta2}$ trans-heterozygote and *gfat2*$^{\Delta1/+}$ control first instar larvae in 5% sucrose (D) and 5% sucrose supplemented with GlcNAc (E). Error bars are standard deviation of the mean from three replicate experiments conducted in parallel.

FIGURE S2 related to figure 2

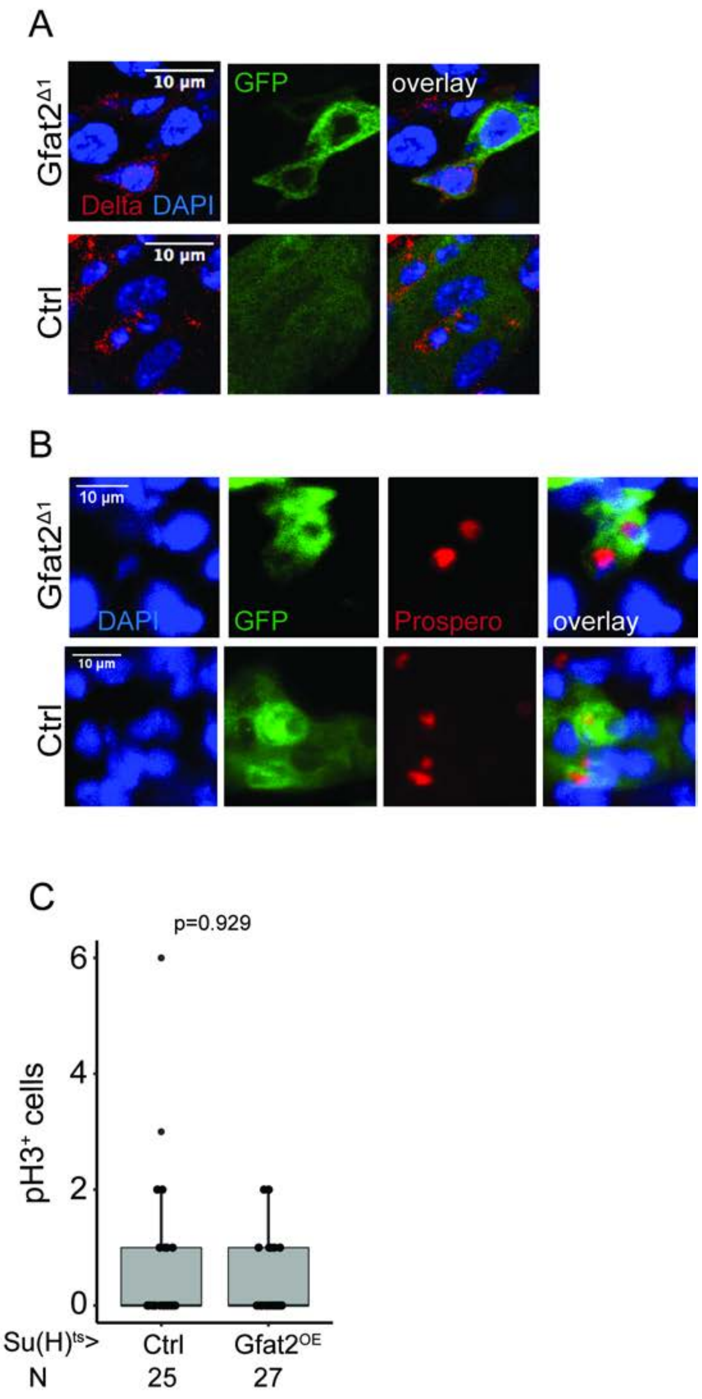

Figure S2. ISCs of the *gfat2$^{\Delta1}$* intestinal clones are viable and able to undergo asymmetric cell divisions and EE lineage differentiation. Related to Figure 2.

(A) *gfat2*$^{\Delta 1}$ and control MARCM clones stained with the ISC marker anti-Delta antibody 14 days after clone induction. (B) *gfat2*$^{\Delta 1}$ and control MARCM clones stained with the EE marker anti-Prospero 7 days after clone induction. (C) Overexpression of Gfat2 by the Su(H)-Gal4^{ts} driver does not change midgut mitotic index. Quantification of the pH3 positive cells from Su(H)-Gal4^{ts}>control and Esg-Gal4^{ts}>UAS-Gfat2 intestines. P-values are calculated by Wilcoxon rank-sum test.

FIGURE S3 related to figure 3

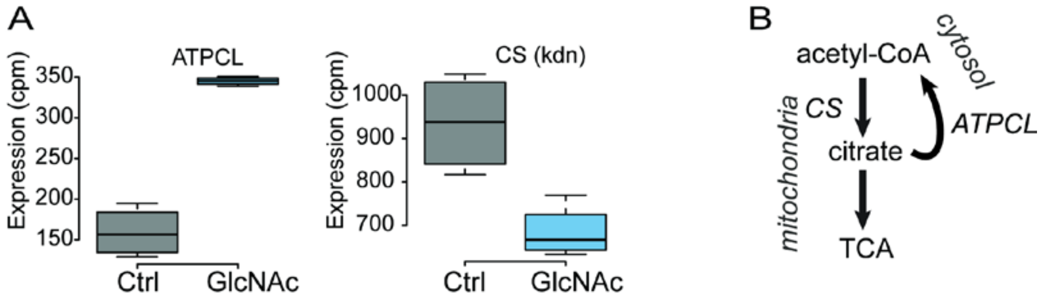

Figure S3. HBP induced transcriptional changes in genes involved in citrate metabolism. Related to Figure 3.

(A) mRNA expressions (counts per million, cpm) of ATP citrate lyase (ATPCL) and Citrate synthase (CS) (kdn) on Ctrl vs. GlcNAc diet. (B) Schematics of the role of CS and ATPCL in citrate metabolism driving the activities of TCA cycle and fatty acid biosynthesis, respectively.

FIGURE S4 related to figure 4

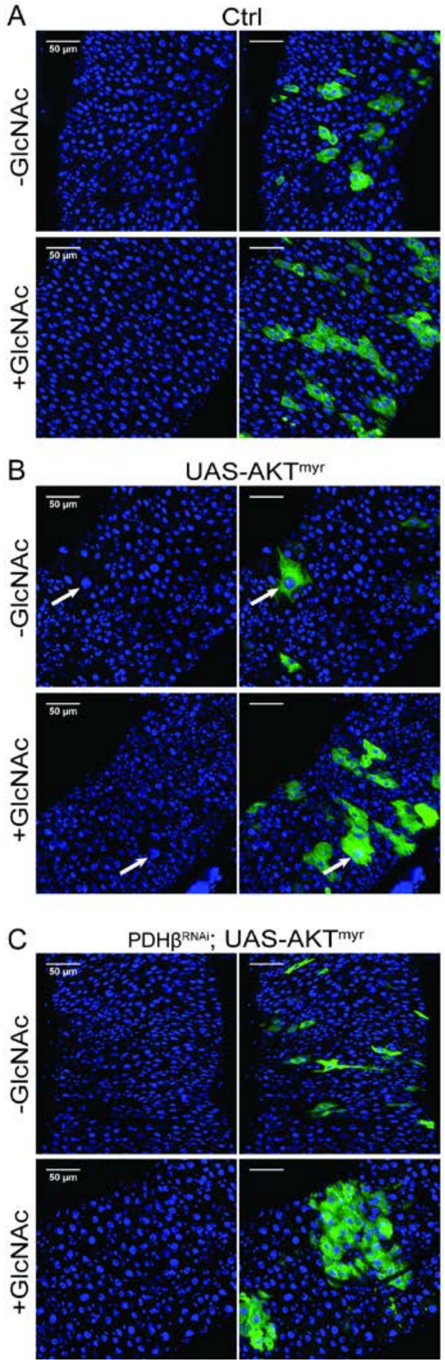

Figure S4. The regulatory output of Akt^{myr} expression in intestinal MARCM clones is determined by dietary GlcNAc and pyruvate metabolism. Related to Figure 4.

Midgut MARCM clones of control (A), UAS-Akt^{myr} (B) and PDHβ^{RNAi}; UAS-Akt^{myr} (C) with or without dietary GlcNAc. Arrows in (B) point to enlarged cells.

Table S3. Number of samples used in the clonal experiments. Related to Figures 1, 2, 3 & 4

Figure	Genotype	Diet	N guts	N clones
1D	MARCM>Ctrl	1x -GlcNAc	12	209
1D	MARCM>Ctrl	1x +GlcNAc	14	224
1D	MARCM>Ctrl	0.25x -GlcNAc	14	209
1D	MARCM>Ctrl	0.25x +GlcNAc	11	146
2F	MARCM>Ctrl	1x -GlcNAc	12	242
2F	MARCM>Gfat2$^{\Delta 1}$	1x -GlcNAc	10	176
2F	MARCM>Gfat2$^{\Delta 1}$	1x +GlcNAc	11	152
2F	MARCM>UAS-Gfat2; Gfat2$^{\Delta 1}$	1x -GlcNAc	7	92
2G	MARCM>Ctrl	1x -GlcNAc	12	216
2G	MARCM>Ctrl	0.25x -GlcNAc	8	136
2G	MARCM>UAS-Gfat2	1x -GlcNAc	14	297
2G	MARCM>UAS-Gfat2	0.25x -GlcNAc	14	252
3F	MARCM>Ctrl	0.25x -GlcNAc	10	217
3F	MARCM>Ctrl	0.25x +GlcNAc	9	136
3F	MARCM>PDHα RNAi	0.25x -GlcNAc	11	187
3F	MARCM>PDHα RNAi	0.25x +GlcNAc	11	177
3F	MARCM>PDHβ RNAi	0.25x -GlcNAc	9	177
3F	MARCM>PDHβ RNAi	0.25x +GlcNAc	9	141
3F	MARCM>LDH RNAi	0.25x -GlcNAc	8	146
3F	MARCM>LDH RNAi	0.25x +GlcNAc	10	169
4A	MARCM>Ctrl	0.25x -GlcNAc	12	210
4A	MARCM>Ctrl	1x -GlcNAc	14	214
4A	MARCM>Gfat2$^{\Delta 1}$	0.25x -GlcNAc	13	224
4A	MARCM>Gfat2$^{\Delta 1}$	1x -GlcNAc	13	251
4D	MARCM>Ctrl	1x -GlcNAc	10	139
4D	MARCM>Ctrl	1x +GlcNAc	10	141
4D	MARCM>UAS-InR^{DN}	1x -GlcNAc	9	184
4D	MARCM>UAS-InR^{DN}	1x +GlcNAc	10	110
4D	MARCM>UAS-InR^{A1325D}	1x -GlcNAc	11	114
4D	MARCM>UAS-InR^{A1325D}	1x +GlcNAc	13	177
4D	MARCM>UAS-InR$^{\alpha del}$	1x -GlcNAc	12	231
4D	MARCM>UAS-InR$^{\alpha del}$	1x +GlcNAc	9	140
4D	MARCM>UAS-Erk^{CA}	1x -GlcNAc	11	152
4D	MARCM>UAS-Erk^{CA}	1x +GlcNAc	13	176
4F	MARCM>Ctrl	1x -GlcNAc	10	248
4F	MARCM>UAS-Akt^{myr}	1x -GlcNAc	10	69
4F	MARCM>UAS-Gfat2; UAS-Akt^{myr}	1x -GlcNAc	10	263
