# Supplementary material for: Stem Cell Intrinsic Hexosamine Metabolism Regulates Intestinal Adaptation to Nutrient Content
Source: Dev Cell. 2018 Oct 8;47(1):112–121.e3. doi: 10.1016/j.devcel.2018.08.011 (PMC6179903; doi:10.1016/j.devcel.2018.08.011)
Supplement: Document S2. Article plus Supplemental Information [file mmc4.pdf]

Short Article

Developmental Cell

Stem Cell Intrinsic Hexosamine Metabolism Regulates Intestinal Adaptation to Nutrient Content

Graphical Abstract

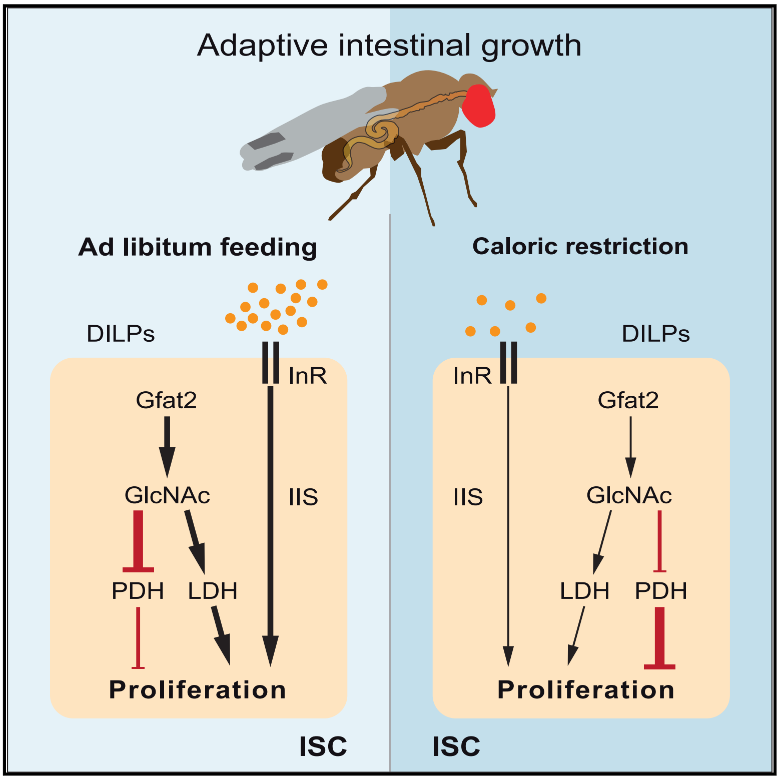

Highlights

- HBP is a mediator of *Drosophila* midgut adaptation to nutrient content
- ISC intrinsic HBP is a necessary and sufficient driver of stem cell divisions
- HBP activity regulates a Warburg-like metabolic reprogramming of the intestine
- HBP activity determines the output of InR signaling of the ISCs

Authors

Jaakko Mattila, Krista Kokki, Ville Hietakangas, Michael Boutros

Correspondence

m.boutros@dkfz-heidelberg.de

In Brief

Intestinal stem cells respond to nutrients in order to maintain tissue homeostasis. Mattila et al. uncover a role for hexosamine biosynthesis pathway as a mechanism of intestinal stem cell nutrient sensing in *Drosophila*. Hexosamine biosynthesis pathway promotes metabolic reprogramming and interacts with insulin signaling to regulate intestinal stem cell proliferation.

Mattila et al., 2018, Developmental Cell *47*, 112–121
October 8, 2018 © 2018 The Author(s). Published by Elsevier Inc.
https://doi.org/10.1016/j.devcel.2018.08.011

CellPress

Developmental Cell
Short Article

Stem Cell Intrinsic Hexosamine Metabolism Regulates Intestinal Adaptation to Nutrient Content

Jaakko Mattila,[1] Krista Kokki,[2,3] Ville Hietakangas,[2,3] and Michael Boutros[1,4,*]
[1]German Cancer Research Center, Division of Signaling and Functional Genomics and Heidelberg University, Heidelberg 69120, Germany
[2]Faculty of Biological and Environmental Sciences, University of Helsinki, Helsinki 00790, Finland
[3]Institute of Biotechnology, University of Helsinki, Helsinki 00790, Finland
[4]Lead Contact
*Correspondence: m.boutros@dkfz-heidelberg.de
https://doi.org/10.1016/j.devcel.2018.08.011

SUMMARY

The intestine is an organ with an exceptionally high rate of cell turnover, and perturbations in this process can lead to severe diseases such as cancer or intestinal atrophy. Nutrition has a profound impact on intestinal volume and cellular architecture. However, how intestinal homeostasis is maintained in fluctuating dietary conditions remains insufficiently understood. By utilizing the *Drosophila* midgut model, we reveal a novel stem cell intrinsic mechanism coupling cellular metabolism with stem cell extrinsic growth signal. Our results show that intestinal stem cells (ISCs) employ the hexosamine biosynthesis pathway (HBP) to monitor nutritional status. Elevated activity of HBP promotes Warburg effect-like metabolic reprogramming required for adjusting the ISC division rate according to nutrient content. Furthermore, HBP activity is an essential facilitator for insulin signaling-induced ISC proliferation. In conclusion, ISC intrinsic hexosamine synthesis regulates metabolic pathway activities and defines the stem cell responsiveness to niche-derived growth signals.

INTRODUCTION

Tissue homeostasis depends on cell turnover replacing aged and damaged cells through asymmetric stem cell divisions. The rate of cell turnover is particularly high in the intestine and regulated by the interaction between the intestinal stem cells (ISCs) and the supportive cellular environment called the niche (Crosnier et al., 2006; Jiang and Edgar, 2012). Since the intestine is a major energy-consuming tissue, modulating intestinal volume and cellular architecture is an important adaptation to fluctuating nutrient availability (Matheson et al., 2000; Mihaylova et al., 2014). For example, reduced calorie intake leads to shorter villi, fewer enterocytes, and reduced overall mass of the small intestine of murine models, and re-feeding reverses these changes (Altmann, 1972; Chappell et al., 2003; Dunel-Erb et al., 2001; Yilmaz et al., 2012). In addition, excess calorie consumption has been shown to have profound implications in the physiology of the intestine, exemplified by epidemiological studies linking obesity and colon cancer incidence (Aleksandrova et al., 2013; Bassett et al., 2010; Comstock et al., 2014). Accordingly, nutrition is an important facilitator of the regulation of the cell turnover rate in the intestine.

Cells respond to nutritional cues through non-cell-autonomous humoral signals such as insulin as well as cell-autonomously through intracellular nutrient sensors such as ChREBP and mTOR signaling (Havula and Hietakangas, 2012; Hietakangas and Cohen, 2009). Furthermore, recent reports show that metabolic pathway activities can be important facilitators of the cellular response to nutrient availability (Mattila et al., 2015; Teesalu et al., 2017; Wellen et al., 2010). ISCs, residing in their prospective niche, are subjected to an additional layer of regulation through niche-secreted factors (Mihaylova et al., 2014). For example, ISC self-renewal in fluctuating dietary conditions is regulated by a cyclic ADP ribose paracrine signal from the Paneth cells of the niche (Yilmaz et al., 2012).

The fruit fly *Drosophila melanogaster* has become a valuable model in understanding the molecular mechanisms guiding the intestinal renewal process (Li and Jasper, 2016; Liang et al., 2017). The fly midgut, a counterpart for the mammalian small intestine, is adaptive to prevailing nutritional conditions. When flies are kept on a calorie-restricted diet, the midgut shrinks in size due to enterocyte apoptosis and attenuated stem cell division rate (Choi et al., 2011; McLeod et al., 2010; O'Brien et al., 2011). Food intake, in turn, results in an expansion of the progenitor cell population and a consequent midgut regeneration. The feeding and fasting cycles are accompanied by changes in local insulin production, and modulating the insulin responsiveness of the ISCs has profound implications to the adaptation of the midgut to nutrient content (Choi et al., 2011; O'Brien et al., 2011). Current knowledge emphasizes the role of ISC extrinsic nutrient-sensing mechanisms, i.e., circulating insulin in regulating the adaptation of the intestine to nutrient availability (Choi et al., 2011; O'Brien et al., 2011). Furthermore, the intestine is a well-established nutrient-sensing organ eliciting systemic signals for inter-organ communication important for the maintenance of organismal homeostasis (Song et al., 2014, 2017). However, if and how ISCs sense nutritional status cell-autonomously

 © 2018 The Author(s). Published by Elsevier Inc.
This is an open access article under the CC BY license (http://creativecommons.org/licenses/by/4.0/).

and how ISC intrinsic nutrient metabolism is linked to extrinsic growth signals has not yet been resolved.

By utilizing the *Drosophila* midgut as a model, we reveal a novel mechanism of ISC regulation integrating the intrinsic signal from the metabolism with extrinsic growth signal. The mechanism translates hexosamine biosynthesis pathway (HBP) activity via a Warburg effect-like regulatory switch in central metabolism into ISC division rate. HBP activity also determines the responsiveness of insulin receptor (InR)-mediated signaling in the ISCs, implying a previously unprecedented control of growth signal interpretation by cell intrinsic metabolic signal. Through the uncovered mechanism, we place HBP as a key player regulating ISC response to nutrition and midgut adaptation.

RESULTS

HBP Is a Mediator of Diet-Dependent Midgut Adaptation

In an attempt to genetically identify mediators of adult fly ISC activation, we uncovered components of HBP to play a role in this process (data not shown). HBP is a nutrient-responsive metabolic pathway, incorporating intracellular glucose, glutamine, acetyl-CoA, and UTP into the synthesis of UDP-GlcNAc, a substrate for macromolecule glycosylation (Figure 1A). When exploring the role of HBP in ISCs, we encountered that feeding flies with an intermediate of HBP, N-acetyl-D-glucosamine (hereafter GlcNAc), promoted ISC proliferation as measured by the propagation of cell number in midgut clones (Figures 1B and 1C). We utilized the number of cells in mosaic analysis with a repressible cell marker (MARCM) clones within the R4c region as a surrogate for midgut adaptation (Figure 1B). We scored midgut clonal cell numbers in either undiluted (1×) or diluted (0.25×, hereafter calorie restriction) fly food. As expected, the cell numbers within the clones were reduced upon calorie restriction. Strikingly, when the calorie-restricted diet was supplemented with 0.1 M GlcNAc, the clone size was sustained at the level of non-calorie-restricted flies. In contrast, in undiluted food, GlcNAc supplementation only modestly increased the clone size (Figures 1C and 1D). To exclude the possibility that flies in the GlcNAc diet have elevated nutrient uptake, we monitored fly feeding by a colorimetric assay (Tennessen et al., 2014). We noticed no increase in food intake in flies kept in the GlcNAc-supplemented food compared to the flies fed in the control diet (Figure 1E). These results show that dietary GlcNAc can maintain midgut clone size during calorie restriction independent of food intake.

HBP Is a Necessary and Sufficient Driver of ISC Divisions

Dietary GlcNAc is taken up by cells through glucose transporters and incorporated into HBP flux (Na et al., 2013; Wellen et al., 2010). Our results suggest that on a calorie-restricting diet, HBP activity is limiting ISC divisions and that upon a full diet, the pathway is already close to saturation or restricted through negative feedback regulation (Traxinger and Marshall, 1991). We next asked whether HBP regulates ISC divisions through cell intrinsic mechanisms by analyzing midgut clones deficient for HBP activity. To this end, we generated loss-of-function mutants of the rate-limiting enzyme in HBP, glutamine fructose-6-phosphate aminotransferase (Gfat) (Marshall et al., 1991). The *Drosophila* genome contains two Gfat homologs, *gfat1* and *gfat2*. According to the published transcriptome of the *Drosophila* midgut cells, *gfat2* is the prevailing isoform expressed in the ISCs (Dutta et al., 2015). By CRISPR/Cas9-mediated genome engineering, we recovered *gfat2*$^{\Delta 1}$ and *gfat2*$^{\Delta 2}$ alleles with 20 and 4 base pair coding region deletions, respectively (Figure 2A). Trans-heterozygote *gfat2*$^{\Delta 1/\Delta 2}$ mutants were first instar lethal but were rescued to pupal stage by an addition of GlcNAc to the fly food (Figures 2B, 2C, and S1). Furthermore, *gfat2*$^{\Delta 1/\Delta 2}$ mutant animals died rapidly on a 5% sucrose diet but were completely rescued by GlcNAc supplementation (Figure S1). Together, these results show that the growth and lethality phenotypes of the *gfat2* mutant animals are due to reduced GlcNAc synthesis and impaired HBP flux.

To address the role of Gfat2 in the fly midgut, we analyzed the proliferation and differentiation of *gfat2*$^{\Delta 1}$ mutant cells in intestinal MARCM clones. ISCs mutant for *gfat2* was viable and able to divide asymmetrically as shown by the presence of the ISC marker Delta-positive cells within *gfat2*$^{\Delta 1}$ clones (Figure S2). In addition, the ISCs mutant for *gfat2* was able to differentiate into the EE lineage, as shown by the presence of the EE cell marker Prospero-positive cells within *gfat2*$^{\Delta 1}$ clones (Figure S2). However, the size of daughter cells arising from *gfat2*$^{\Delta 1}$ progenitors was smaller than cells in control clones. Hence, we stained *gfat2*$^{\Delta 1}$ clones for anti-Pdm1, a marker of mature enterocytes, and noticed that most of the cells lacked noticeable Pdm1 expression, indicating that these cells are defective in enterocyte differentiation (Figure 2D). Moreover, the cells in the *gfat2*$^{\Delta 1}$ clones proliferated in a significantly lowered rate compared to wild-type clones (Figures 2E and 2F). Remarkably, the clonal propagation phenotype of *gfat2*$^{\Delta 1}$ clones was completely rescued by dietary GlcNAc or by exogenous expression of Gfat2 (Figures 2E and 2F). The results indicate that the HBP flux and the production of hexosamines are restored in the *gfat2*$^{\Delta 1}$ ISCs by dietary GlcNAc. We then asked if increased hexosamine synthesis is sufficient for ISC activation. To this end, we overexpressed the Gfat2 enzyme in midgut clones and measured the clone size propagation. Similar to the results obtained from GlcNAc supplementation experiments, Gfat2 overexpression resulted in an increased net clone size, and the phenotype was pronounced in calorie-restricted flies (Figure 2G). Finally, to ask if HBP is a stem cell-autonomous inducer of cell division, we overexpressed Gfat2 by the Esg-Gal4 driver, which is expressed in the stem cells and the enteroblast progenitors (Micchelli and Perrimon, 2006), and counted pH3-positive cells as a readout of the ISC division rate. Overexpression of Gfat2 in the Esg^{+} cells led to a noticeable increase in the pH3-positive cells, whereas overexpression by the enteroblast-specific Su(H)-Gal4 driver had no effect (Figures 2H and S2). Taken together, the results presented above show that HBP activity is a necessary and sufficient regulator of ISC division. In addition, HBP is required for daughter cell growth and enterocyte maturation.

HBP Mediates ISC Activation through Regulation of Pyruvate Metabolism

In the search for a mechanism of the HBP-mediated ISC activation, we performed RNA sequencing (RNA-seq) gene expression

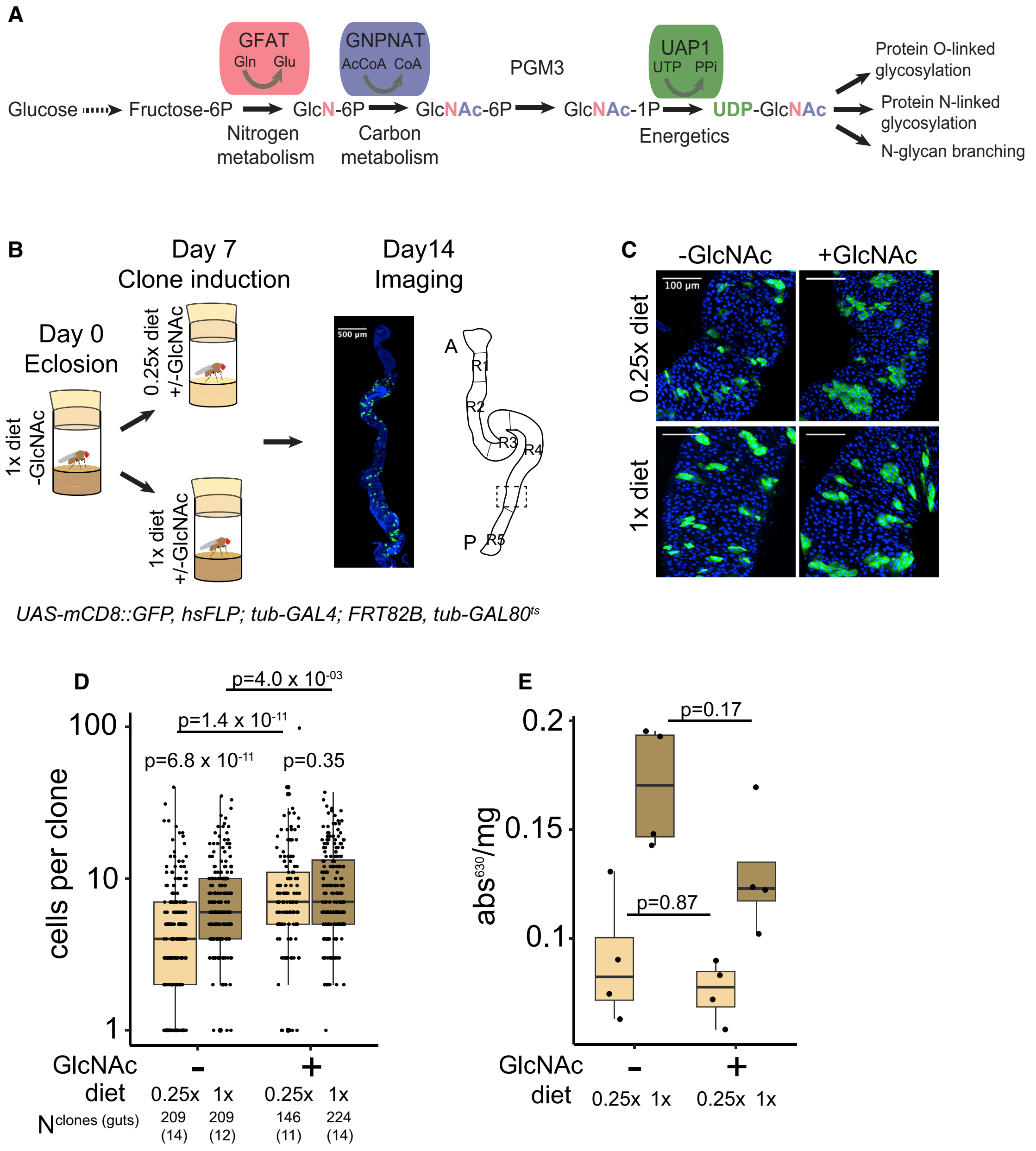

Figure 1. HBP Is a Mediator of Diet-Dependent Midgut Adaptation

(A) Schematics of the hexosamine biosynthesis pathway (HBP). In this study, we fed flies with GlcNAc-supplemented food to stimulate HBP flux. GlcNAc is taken up by glucose transporters and enters the pathway after phosphorylation by N-acetylglucosamine kinase to yield GlcNAc-6P. HBP integrates inputs from glucose, glutamine (nitrogen metabolism), acetyl-CoA (carbon metabolism), and UTP (energy metabolism), making it a sensor of cellular nutrient and energy metabolism.

(B) Schematics of the experimental setup employed in the study.

(C) Dietary GlcNAc promotes midgut growth of calorie-restricted flies. Wild-type MARCM clones in the control diet (1×) and calorie-restricted diet (0.25×) supplemented with GlcNAc (0.1 M).

(D) Quantification of (C).

(E) Quantification of adult fly nutrient uptake by a colorimetric assay from dietary conditions and genotype in (C). The experiment was performed in quadruplicate with pools of eight flies per replicate. p values in (D) are calculated by Wilcoxon rank-sum test with multiple testing correction (false discovery rate < 0.05). p values in (E) are calculated by two-way ANOVA, followed by post hoc Tukey HSD test. The number of samples in the clonal experiments are indicated in the figure and in Table S3.

profiling analysis from intestines of calorie-restricted flies exposed to dietary GlcNAc. From the dataset, we performed differentially expressed gene analysis (DEG) and gene set enrichment (GSE) analysis (Tables S1 and S2). Notably, the results revealed that dietary GlcNAc comprehensively inhibits the expression of genes involved in the TCA cycle and oxidative

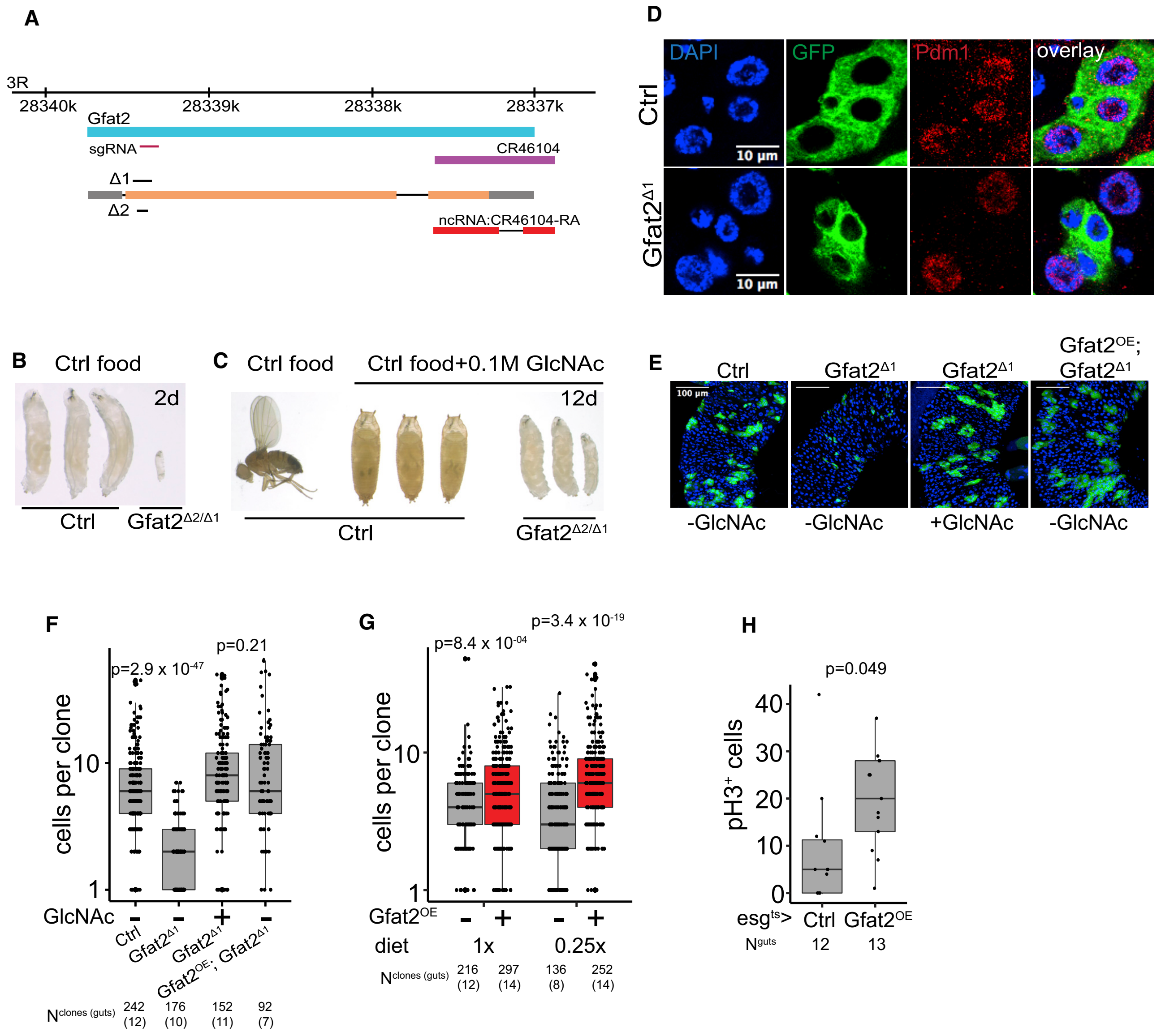

Figure 2. Gfat2 Is an ISC Autonomous Regulator of Cell Division and Cell Growth

(A) Schematics of the genomic location of the *gfat2* gene and the recovered alleles used in this study.

(B) *gfat2* null animals are first instar lethal but rescued by dietary GlcNAc. $gfat2^{\Delta 1/\Delta 2}$ trans-heterozygote and wild-type controls 2 days after hatching in control diet.

(C) Wild-type control 12 days after hatching in control diet (adult fly in left) and $gfat2^{\Delta 1/\Delta 2}$ trans-heterozygote and control animals in a diet supplemented with 0.1 M GlcNAc (pupae and larvae on right).

(D) Cells of the $gfat2^{\Delta 1}$ intestinal clones are growth defective and lack an enterocyte marker. Control (upper inset) and $gfat2^{\Delta 1}$ (lower inset) MARCM clones stained with the enterocyte marker anti-Pdm1 antibody.

(E) Intestinal $gfat2^{\Delta 1}$ clones are growth defective and rescued by dietary GlcNAc. MARCM clones of control -GlcNAc, $gfat2^{\Delta 1}$ -GlcNAc, $gfat2^{\Delta 1}$ +GlcNAc, and UAS-Gfat2; $gfat2^{\Delta 1}$ -GlcNAc.

(F) Quantification of (E).

(G) Intestinal MARCM clones overexpressing Gfat2 are larger than controls due to increased cell numbers. Quantification of cell numbers in control and UAS-Gfat2 MARCM clones in the control diet (1×) and calorie-restricted diet (0.25×).

(H) Overexpression of Gfat2 by the Esg-Gal4^{ts} driver leads to an increased midgut mitotic index. Quantification of the pH3-positive cells from Esg-Gal4^{ts}>control and Esg-Gal4^{ts}>UAS-Gfat2 intestines. p values in (F) and (G) are calculated by Wilcoxon rank-sum test with multiple testing correction (FDR < 0.05). p values in (H) are calculated by Wilcoxon rank-sum test. The number of samples in the clonal experiments are indicated in the figure and in Table S3.

See also Figures S1 and S2.

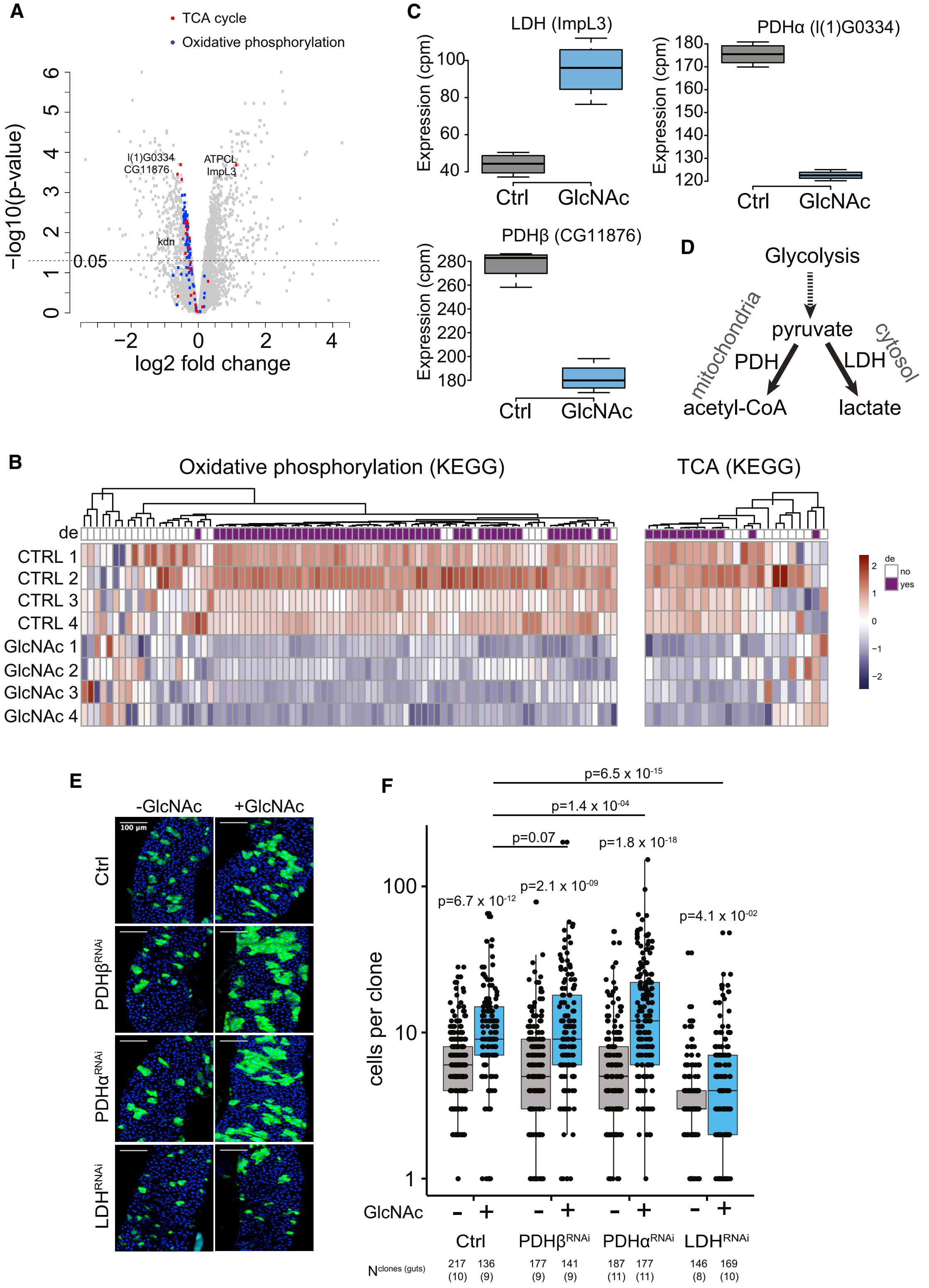

Figure 3. ISC Activation through HBP Is Mediated by Pyruvate Metabolism

(A) Volcano plot showing the global gene expression changes in midgut cells after GlcNAc feeding. Genes involved in the TCA cycle (KEGG) (red dots) and oxidative phosphorylation (KEGG) (blue dots) are shown.

(B) Heatmaps showing gene expression changes of all expressed genes annotated for TCA cycle (KEGG) and oxidative phosphorylation (KEGG) in midgut cells after GlcNAc feeding. DE sidebar denotes differential expression (violet bar for differentially expressed).

(legend continued on next page)

phosphorylation in mitochondria (Figures 3A and 3B). This observation indicates that the midgut exhibits a metabolic switch from respirative metabolism to glycolysis upon HBP activation resembling the so-called Warburg effect, which produces metabolic precursors necessary for the rapidly proliferating cells (Kroemer and Pouyssegur, 2008; Lu et al., 2015). In this condition, the end product of glycolysis, pyruvate, is further metabolized by lactate dehydrogenase (LDH) to yield lactate. Indeed, the sole fly ortholog of LDH (*ImpL3*) was strongly upregulated, whereas the mitochondrial enzyme pyruvate dehydrogenase (*PDH*α/β, CG11876, and *l(1)G0334*), driving the conversion of pyruvate to acetyl-CoA and oxidative phosphorylation, was downregulated in our dataset (Figures 3A–3C). Our data also show that the enzyme citrate synthase (*CS*, *kdn*), catalyzing the conversion of acetyl-CoA and oxaloacetate to citrate as the first step of the TCA cycle was downregulated, whereas the cytoplasmic ATP citrate lyase (*ATPCL*) was upregulated upon dietary GlcNAc (Figures 3A and S3). These findings indicate that HBP regulates a gene expression program for the conversion of acetyl-CoA to fatty acids as opposed to their utilization for energy generation in oxidative phosphorylation. Taken together, these results support a model where, under the condition of elevated HBP activity, the midgut cells possess increased Warburg-like metabolism.

A recent study shows that mitochondrial pyruvate metabolism regulates ISC proliferation in the mouse and fly models (Schell et al., 2017). Specifically, it was shown that knockdown of the LDH and PDH enzymes either decreased or increased ISC divisions in the fly midgut, respectively. Hence, we asked if the HBP-mediated ISC activation is due to altered pyruvate metabolism. To this end, we generated LDH and PDH knockdown MARCM clones and followed the clonal propagation in the presence of dietary GlcNAc. When kept in the calorie-restricted diet, PDH knockdown had no effect on the clonal cell numbers, whereas the LDH knockdown clones were slightly smaller (Figures 3D and 3E). However, when the diet was supplemented with GlcNAc to stimulate HBP flux and ISC activation, we observed a synergistic effect between HBP and PDH knockdown resulting in larger clone size compared to the controls. In contrast, in the LDH knockdown clones, the growth-promoting effect of the dietary GlcNAc was nearly completely abolished (Figures 3D and 3E). These results suggest that pyruvate metabolism, and more specifically the production of lactate by the LDH, is a key step in the HBP-mediated ISC activation (Figure 3D).

HBP Is an Essential Facilitator of InR Signaling-Mediated Midgut Growth

The results presented above and the previously known role of HBP as a nutrient-responsive pathway suggest a model in which dynamic HBP activity is a mechanism to transmit information about ISC intrinsic nutritional and energetic status resulting in midgut adaptation. To test this idea directly, we asked if HBP activity is required for midgut adaptation in response to nutrients. Hence, we monitored cell propagation of *gfat2*$^{\Delta 1}$ mutant clones in a calorie-restricted versus non-restricted diet. Indeed, nutrient content had no effect on the size of *gfat2*$^{\Delta 1}$ mutant clones, indicating that HBP activity is required for adjusting the ISC division rate to the prevailing nutrient content (Figure 4A).

Since the adaptation of the midgut to fluctuating dietary conditions is mediated by systemic insulin signal (IIS) emanating from the midgut visceral muscle and brain insulin-producing cells (Figure 4B) (O'Brien et al., 2011), we next asked if the HBP-mediated ISC activation interacts with InR signaling. To this end, we first generated intestinal MARCM clones expressing a dominant negative InR (InR^{DN}). In the control non-diluted diet, the inhibition of InR signaling resulted in a significantly impaired growth of the midgut clones. Strikingly, dietary GlcNAc completely rescued the InR^{DN} phenotype (Figures 4C and 4D). This observation suggests the existence of an InR signaling-independent compensatory growth mechanism through HBP, relying solely on the stem cell intrinsic nutritional status. To further elucidate the interaction between InR signaling and HBP, we generated midgut clones expressing activated InR (InR^{CA}; InR^{A1325D} and InR$^{\alpha del}$). Overexpressing the InR^{A1325D} variant in midgut stem cell clones has previously been shown to either increase or decrease clonal growth (Choi et al., 2011; O'Brien et al., 2011). When flies were fed in our control non-diluted diet, we found that midgut clones expressing the activated InR variants were smaller in size compared to the control clones (Figures 4C and 4D). However, when the diet was supplemented with GlcNAc, the clone size was dramatically increased, exceeding the level of the control clones in GlcNAc-fed animals (Figures 4C and 4D). In contrast, dietary GlcNAc did not further increase the size of MARCM clones expressing an activated variant of Erk (Erk^{CA}), suggesting that HBP interacts specifically with IIS (Figures 4C and 4D).

To further explore the interaction between HBP and IIS, we generated MARCM clones expressing an activated variant of Akt (Akt^{myr}), a well-known downstream effector of InR, and monitored the intestinal clonal growth in co-expression with Gfat2 or with dietary GlcNAc. Overexpressing Akt^{myr} resulted in striking shrinkage of the clone size measured by cell numbers (Figures 4E and 4F). Even though clonal cell numbers were reduced in the Akt^{myr}-overexpressing clones, the cell size was clearly increased (Figure S4), a phenotype previously reported in intestinal clones overexpressing InR (Choi et al., 2011). Strikingly, when co-expressed with Gfat2 or in the presence of dietary GlcNAc, Akt^{myr}-overexpressing clones were rescued as measured by cell numbers, and knockdown of PDH augmented this phenotype (Figures 4E, 4F, and S4). In conclusion, the results presented show that HBP activity determines the regulatory output of InR-Akt signaling in ISCs.

(C) mRNA expressions (counts per million, cpm) of lactate dehydrogenase (*LDH*) and pyruvate dehydrogenase (*PDH*α/β) on control versus GlcNAc diet.

(D) Schematics of the role of PDH and LDH in pyruvate metabolism driving pyruvate conversion to acetyl-CoA and lactate, respectively.

(E) Modulating pyruvate metabolism through PDH and LDH knockdowns alters ISC responsiveness to dietary GlcNAc. MARCM clones of control, PDHα^{RNAi}, PDHβ^{RNAi}, and LDH^{RNAi} in 0.25× calorie-restricted diet with GlcNAc supplementation.

(F) Quantification of (D). p values in (E) are calculated by Wilcoxon rank-sum test with multiple testing correction (FDR $<$ 0.05). The number of samples in the clonal experiments are indicated in the figure and in Table S3.

See also Figure S3 and Tables S1 and S2.

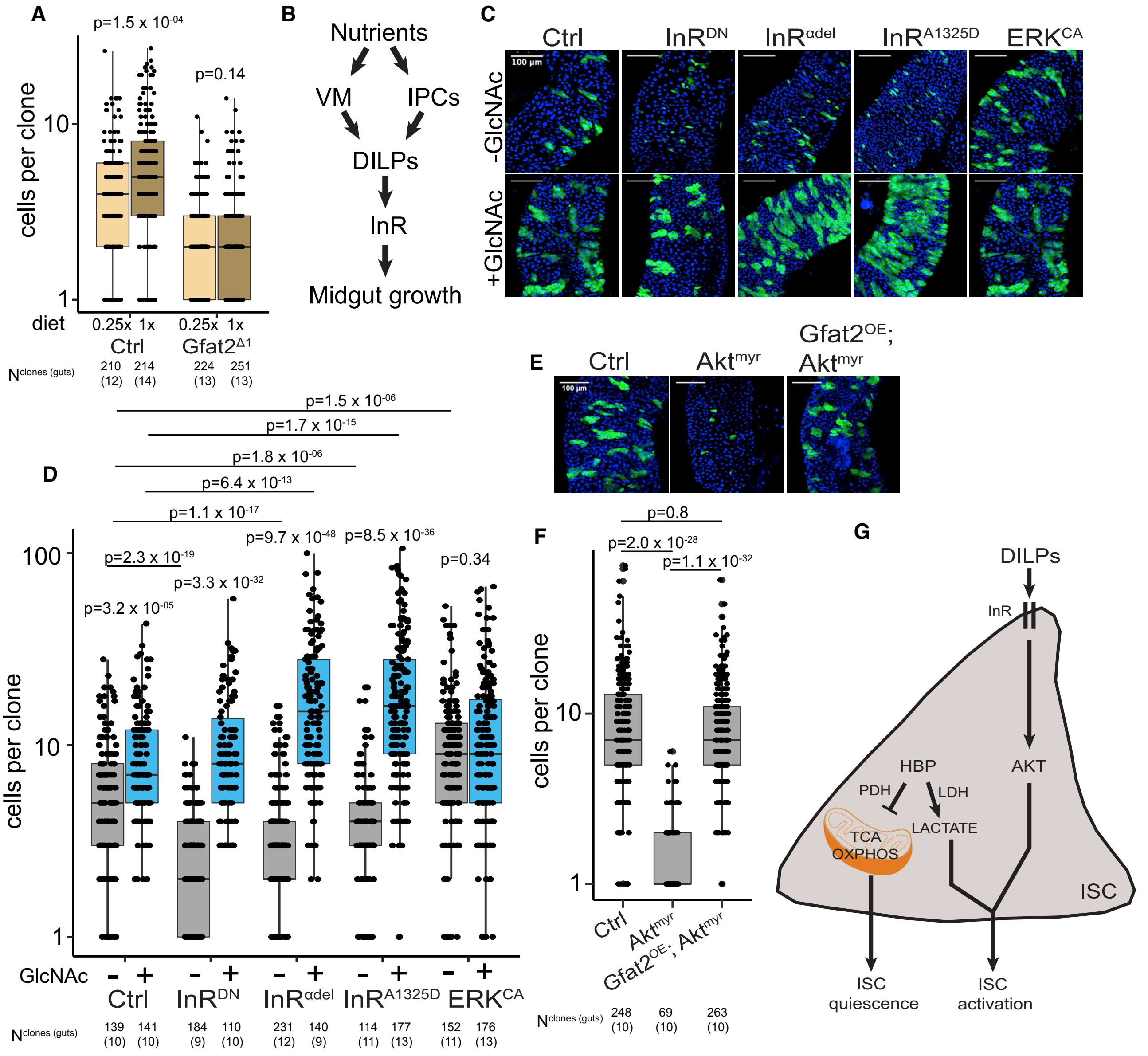

Figure 4. HBP Is an Essential Facilitator of InR-Mediated ISC Proliferation

(A) Gfat2 is necessary for nutrient-dependent midgut adaptation. Quantification of the cell numbers in control and *gfat2*$^{\Delta 1}$ MARCM clones in the control diet (1×) and calorie-restricted diet (0.25×).

(B) Schematics of the role of nutrients in ISC extrinsic control of midgut growth. Feeding elicits local insulin (DILPs) production from visceral muscle (VM) and brain insulin-producing cells (IPC). Insulin activates the ISC insulin receptor (InR) signaling leading to ISC activation to cell growth and division.

(C) HBP is an essential facilitator of InR signaling-mediated ISC proliferation. MARCM clones of control, InR^{DN}, InR^{CA} (InR$^{\alpha del}$ & InR^{A1325D}), and Erk^{CA} in the absence or presence of dietary GlcNAc in control 1× diet.

(D) Quantification of (C).

(E) HBP is an essential facilitator of Akt-mediated ISC proliferation. Control, Akt^{myr}, and Gfat2; Akt^{myr} expressing MARCM clones in control 1× diet.

(F) Quantification of (E).

(G) A model deciphering the role of HBP in ISC activation. HBP activity regulates the balance between oxidative phosphorylation and glycolysis-mediated lactate production and ISC quiescence and activation, respectively. p values in (A), (D), and (F) are calculated by Wilcoxon rank-sum test with multiple testing correction (FDR < 0.05). The number of samples in the clonal experiments are indicated in the figure and in Table S3.

See also Figure S4.

DISCUSSION

Nutrition has been recognized as a key modulator of intestinal physiology, size, and morphology (Mihaylova et al., 2014; Shaw, 2012; Yilmaz et al., 2012). In addition, ISCs have been shown to respond to specific dietary nutrients such as the amino acids glutamate and methionine (Deng et al., 2015; Obata et al., 2018). Yet, how the fluctuating dietary conditions translate into

the maintenance of intestinal homeostasis remains poorly understood. In this study, we show that *Drosophila* ISCs employ a cell intrinsic nutrient-sensing mechanism dependent on HBP activity to adjust the rate of cell division into the prevailing nutrient content.

The contribution of HBP to cellular processes through N- and O-linked protein glycosylation is well established (Ferrer et al., 2016; Molinari, 2007). UDP-GlcNAc is implicated in diverse cellular processes depending on the cell type and developmental stage. These include, for example, hyaluronic acid production in connective tissue (Oikari et al., 2016), regulation of protein function through O-glycosylation in adipocyte differentiation (Hsieh et al., 2012), hepatocyte insulin responsiveness (Yang et al., 2008), and β cell function (Alejandro et al., 2015). However, how HBP activity contributes to tissue-specific functions in preserving organismal homeostasis is less well understood. For example, increased levels of cellular D-glucosamine (GlcN) were shown to mimic a low-carbohydrate diet in mouse and in *C. elegans*, elevating the lifespan of these model organisms (Weimer et al., 2014). In flies, however, an increased HBP flux through GlcN feeding leads to cardiomyopathy and elevated mortality (Na et al., 2013). In our experiments, we have shown that HBP in the fly intestine regulates the balance between oxidative phosphorylation and glycolysis, contributing to the proliferation of the ISCs and intestinal adaptation to nutrient content. Such metabolic rewiring, also known as the Warburg effect, is a recurrent theme in highly proliferating cancer cells and has also recently been associated with stem cell activation in the mouse and fly models (Flores et al., 2017; Schell et al., 2017). An outstanding question is if the rewiring of energy metabolism is an active driver of ISC activation or merely a passive consequence of it. Accordingly, the mechanism of HBP-mediated metabolic rewiring is not known. HBP could achieve this through several distinct mechanisms, such as by regulating signaling activities through protein O- and/or N-linked glycosylation. For example, proliferation of HBP-dependent hematopoietic cells relies on N-linked glycosylation and cell surface expression of IL-3 receptor α (Wellen et al., 2010). Alternatively, direct mechanisms altering metabolic fluxes of other glucose-metabolizing pathways could play a role. Finally, other cell non-autonomous mechanisms might contribute to ISC activation. Our transcriptional profiling experiment was performed from whole midguts, containing all intestinal cell types, and therefore, we cannot rule out the role of additional non-cell-autonomous mechanisms as shown in the mouse model by the secretion of lactate from Paneth cells to support ISC function (Rodríguez-Colman et al., 2017).

Systemic activation of the InR and the downstream Akt-TSC1/2-TOR signaling branch is triggered by local insulin secretion as a result of feeding (Teleman, 2009). TOR signaling responds also to intracellular amino acid levels, and together with other targets of the InR signaling, TOR regulates cellular growth and entry into mitosis (Saxton and Sabatini, 2017). However, in fly ISCs, TOR is not sufficient to trigger cell divisions. Augmenting TOR activity through inhibition of the negative regulator TSC1/2 leads to ISC withdrawal from the cell cycle without self-renewal and subsequent ISC loss (Amcheslavsky et al., 2011; Kapuria et al., 2012; Quan et al., 2013). These results highlight the need to maintain optimal TOR signaling to assure proper stem cell growth and maintenance, yet additional mechanisms are required to drive ISC divisions. Interestingly, while InR, upstream of TOR, has been shown to be necessary for the *Drosophila* ISC divisions in genetic loss-of-function experiments, gain of function of InR signaling has revealed conflicting results (Amcheslavsky et al., 2011; Choi et al., 2011; Kapuria et al., 2012; O'Brien et al., 2011). Namely, in different experimental settings, expressing a gain-of-function variant of InR in fly intestinal clones results in either increased or attenuated growth rate. Thus, mechanisms modulating the InR signaling responsiveness appear necessary for nutrient-dependent ISC proliferation. In our experimental conditions, activated InR signaling resulted in reduced intestinal clone size as measured by cell numbers. Upon elevated HBP flux, the stem cell divisions within InR^{CA}-expressing clones were greatly enhanced. The result and previous findings by others suggest that on low ISC intrinsic HBP activity, activated InR signaling promotes ISC growth, cessation of cell divisions, and subsequent ISC loss. In order to stimulate ISC division, additional stem cell activation is required through the HBP-mediated metabolic rewiring (Figure 4E). Such regulation highlights the central role of stem cell intrinsic nutrient sensing through HBP, positioning it as an essential facilitator of growth factor-mediated ISC activation. The interdependency of HBP and InR signaling possibly reflects a mechanism protecting the organism from unrestrained stem cell division.

In this study, we have elucidated a mechanism of activating ISCs from calorie-restriction-induced slow division rate, and this finding holds possible therapeutic significance. Interestingly, it has been long recognized that glutamine improves intestine structure and function of murine intestinal atrophy models, i.e., shortened intestinal epithelium or erosion of intestinal villi, or critically ill human patients in parenteral feeding or chemotherapy (Braga-Neto et al., 2008; van der Hulst et al., 1993; Inoue et al., 1993; Klimberg et al., 1990; Miller, 1999). Glutamine is a critical component of the cataplerotic TCA cycle flux, thereby increasing the production of important metabolic intermediates for growth, but also contributes to the activity of the HBP as a substrate for the rate-limiting enzyme Gfat (Owen et al., 2002). Our results from the *Drosophila* intestine model show that Gfat is an essential gatekeeper of nutrient-induced ISC activation. In addition, we show that N-acetyl-D-glucosamine, a widely used dietary supplement, is sufficient to enhance ISC divisions in a calorie restriction-induced intestinal atrophy model. In summary, our findings may offer tools to increase the efficacy of therapies related to recovery from intestinal atrophy.

STAR★METHODS

Detailed methods are provided in the online version of this paper and include the following:

- KEY RESOURCES TABLE
- CONTACT FOR REAGENT AND RESOURCE SHARING
- EXPERIMENTAL MODEL AND SUBJECT DETAILS
  - *Drosophila* Stocks
- METHOD DETAILS
  - MARCM Analysis
  - Food Intake Measurement
  - Generation of Drosophila Mutants

CellPress

- Pupation Curves and Lethality Measurement
- Immunohistochemistry
- RNA-Seq and Data Analysis
- DATA AND SOFTWARE AVAILABILITY
- QUANTIFICATION AND STATISTICAL ANALYSIS

SUPPLEMENTAL INFORMATION

Supplemental Information includes four figures and three tables and can be found with this article online at https://doi.org/10.1016/j.devcel.2018.08.011.

ACKNOWLEDGMENTS

The authors thank W. Chia for the anti-Pdm1 antibody; Bloomington stock center and VDRC for fly strains; DKFZ Light Microscopy Facility for the help with imaging; Dorothee Childs and Liam Childs for the help with statistical analysis; and Paula Jouhten, Fillip Port, Tianzuo Zhan, and Jun Zhou for critically commenting on the manuscript. This work was supported by the Sigrid Juselius Foundation (J.M.), Jane & Aatos Erkko Foundation (V.H.), Academy of Finland (286767 and 312439 to V.H.), and Novo Nordisk Foundation (V.H.). Work in the lab of M.B. is in part supported by an ERC Advanced Grant. M.B. is a member of the Excellence Cluster CellNetworks.

AUTHOR CONTRIBUTIONS

J.M. and M.B. conceived and designed this project. J.M. designed and performed the experiments. All authors analyzed the data. J.M. and M.B. wrote the manuscript. All authors have contributed to the discussion of the results and comments on the manuscript.

DECLARATION OF INTERESTS

The authors declare no competing interests.

Received: December 15, 2017
Revised: July 7, 2018
Accepted: August 14, 2018
Published: September 13, 2018

REFERENCES

Alejandro, E.U., Bozadjieva, N., Kumusoglu, D., Abdulhamid, S., Levine, H., Haataja, L., Vadrevu, S., Satin, L.S., Arvan, P., and Bernal-Mizrachi, E. (2015). Disruption of O-linked N-Acetylglucosamine signaling induces ER stress and β cell failure. Cell Rep. *13*, 2527–2538.

Aleksandrova, K., Pischon, T., Buijsse, B., May, A.M., Peeters, P.H., Bueno-de-Mesquita, H.B., Jenab, M., Fedirko, V., Dahm, C.C., Siersema, P.D., et al. (2013). Adult weight change and risk of colorectal cancer in the European Prospective Investigation into Cancer and Nutrition. Eur. J. Cancer *49*, 3526–3536.

Altmann, G.G. (1972). Influence of starvation and refeeding on mucosal size and epithelial renewal in the rat small intestine. Am. J. Anat. *133*, 391–400.

Amcheslavsky, A., Ito, N., Jiang, J., and Ip, Y.T. (2011). Tuberous sclerosis complex and Myc coordinate the growth and division of Drosophila intestinal stem cells. J. Cell Biol. *193*, 695–710.

Bassett, J.K., Severi, G., English, D.R., Baglietto, L., Krishnan, K., Hopper, J.L., and Giles, G.G. (2010). Body size, weight change, and risk of colon cancer. Cancer Epidemiol. Biomarkers Prev. *19*, 2978–2986.

Braga-Neto, M.B., Warren, C.A., Oriá, R.B., Monteiro, M.S., Maciel, A.A.S., Brito, G.A.C., Lima, A.A.M., and Guerrant, R.L. (2008). Alanyl-glutamine and glutamine supplementation improves 5-fluorouracil-induced intestinal epithelium damage in vitro. Dig. Dis. Sci. *53*, 2687–2696.

Buchon, N., Osman, D., David, F.P.A., Fang, H.Y., Boquete, J.P., Deplancke, B., and Lemaitre, B. (2013). Morphological and molecular characterization of adult midgut compartmentalization in drosophila. Cell Rep. *3*, 1725–1738.

Chappell, V.L., Thompson, M.D., Jeschke, M.G., Chung, D.H., Thompson, J.C., and Wolf, S.E. (2003). Effects of incremental starvation on gut mucosa. Dig. Dis. Sci. *48*, 765–769.

Choi, N.H., Lucchetta, E., and Ohlstein, B. (2011). Nonautonomous regulation of Drosophila midgut stem cell proliferation by the insulin-signaling pathway. Proc. Natl. Acad. Sci. USA *108*, 18702–18707.

Comstock, S.S., Hortos, K., Kovan, B., McCaskey, S., Pathak, D.R., and Fenton, J.I. (2014). Adipokines and obesity are associated with colorectal polyps in adult males: A cross-sectional study. PLoS One *9*, e85939.

Crosnier, C., Stamataki, D., and Lewis, J. (2006). Organizing cell renewal in the intestine: stem cells, signals and combinatorial control. Nat. Rev. Genet. *7*, 349–359.

Deng, H., Gerencser, A.A., and Jasper, H. (2015). Signal integration by Ca(2+) regulates intestinal stem-cell activity. Nature *528*, 212–217.

Dunel-Erb, S., Chevalier, C., Laurent, P., Bach, A., Decrock, F., and Le Maho, Y. (2001). Restoration of the jejunal mucosa in rats refed after prolonged fasting. Comp. Biochem. Physiol. A Mol. Integr. Physiol. *129*, 933–947.

Dutta, D., Dobson, A.J., Houtz, P.L., Gläßer, C., Revah, J., Korzelius, J., Patel, P.H., Edgar, B.A., and Buchon, N. (2015). Regional cell-specific transcriptome mapping reveals regulatory complexity in the adult Drosophila Midgut. Cell Rep. *12*, 346–358.

Ferrer, C.M., Sodi, V.L., and Reginato, M.J. (2016). O-GlcNAcylation in cancer biology: linking metabolism and signaling. J. Mol. Biol. *428*, 3282–3294.

Flores, A., Schell, J., Krall, A.S., Jelinek, D., Miranda, M., Grigorian, M., Braas, D., White, A.C., Zhou, J.L., Graham, N.A., et al. (2017). Lactate dehydrogenase activity drives hair follicle stem cell activation. Nat. Cell *19*, 1017–1026.

Havula, E., and Hietakangas, V. (2012). Glucose sensing by ChREBP/MondoA-Mlx transcription factors. Semin. Cell Dev. Biol. *23*, 640–647.

Hietakangas, V., and Cohen, S.M. (2009). Regulation of tissue growth through nutrient sensing. Annu. Rev. Genet. *43*, 389–410.

Hsieh, T.J., Lin, T., Hsieh, P.C., Liao, M.C., and Shin, S.J. (2012). Suppression of glutamine:fructose-6-phosphate amidotransferase-1 inhibits adipogenesis in 3T3-L1 adipocytes. J. Cell. Physiol. *227*, 108–115.

Inoue, Y., Grant, J.P., and Snyder, P.J. (1993). Effect of glutamine-supplemented Total Parenteral Nutrition on recovery of the small intestine after starvation atrophy. JPEN J. Parenter. Enteral Nutr. *17*, 165–170.

Jiang, H., and Edgar, B.A. (2009). EGFR signaling regulates the proliferation of Drosophila adult midgut progenitors. Development *136*, 483–493.

Jiang, H., and Edgar, B.A. (2012). Intestinal stem cell function in Drosophila and mice. Curr. Opin. Genet. Dev. *22*, 354–360.

Kapuria, S., Karpac, J., Biteau, B., Hwangbo, D., and Jasper, H. (2012). Notch-mediated suppression of TSC2 expression regulates cell differentiation in the drosophila intestinal stem cell lineage. PLoS Genet. *8*, e1003045.

Klimberg, V.S., Salloum, R.M., Kasper, M., Plumley, D.A., Dolson, D.J., Hautamaki, R.D., Mendenhall, W.R., Bova, F.C., Bland, K.I., and Copeland, E.M. (1990). Oral glutamine accelerates healing of the small intestine and improves outcome after whole abdominal radiation. Arch. Surg. *125*, 1040–1045.

Kroemer, G., and Pouyssegur, J. (2008). Tumor cell metabolism: cancer's Achilles' heel. Cancer Cell *13*, 472–482.

Li, H., and Jasper, H. (2016). Gastrointestinal stem cells in health and disease: from flies to humans. Dis. Model. Mech. *9*, 487–499.

Liang, J., Balachandra, S., Ngo, S., and O'Brien, L.E. (2017). Feedback Regulation of Steady-State Epithelial Turnover and Organ Size (Nature Publishing).

Lu, J., Tan, M., and Cai, Q. (2015). The Warburg effect in tumor progression: mitochondrial oxidative metabolism as an anti-metastasis mechanism. Cancer Lett. *356*, 156–164.

Marshall, S., Bacote, V., and Traxinger, R.R. (1991). Discovery of a metabolic pathway mediating glucose-induced desensitization of the glucose transport system. Role of hexosamine biosynthesis in the induction of insulin resistance. J. Biol. Chem. *266*, 4706–4712.

Matheson, P.J., Wilson, M.A., and Garrison, R.N. (2000). Regulation of intestinal blood flow. J. Surg. Res. *93*, 182–196.

Mattila, J., Havula, E., Suominen, E., Teesalu, M., Surakka, I., Hynynen, R., Kilpinen, H., Väänänen, J., Hovatta, I., Käkelä, R., et al. (2015). Mondo-Mlx mediates organismal sugar sensing through the gli-similar transcription factor Sugarbabe. Cell Rep. *13*, 350–364.

McLeod, C.J., Wang, L., Wong, C., and Jones, D.L. (2010). Stem cell dynamics in response to nutrient availability. Curr. Biol. *20*, 2100–2105.

Micchelli, C.A., and Perrimon, N. (2006). Evidence that stem cells reside in the adult Drosophila midgut epithelium. Nature *439*, 475–479.

Mihaylova, M.M., Sabatini, D.M., and Yilmaz, Ö.H. (2014). Dietary and metabolic control of stem cell function in physiology and cancer. Cell Stem Cell *14*, 292–305.

Miller, A.L. (1999). Therapeutic considerations of L-glutamine: a review of the literature. Altern. Med. Rev. J. Clin. Ther. *4*, 239–248.

Molinari, M. (2007). N-glycan structure dictates extension of protein folding or onset of disposal. Nat. Chem. Biol. *3*, 313–320.

Na, J., Musselman, L.P., Pendse, J., Baranski, T.J., Bodmer, R., Ocorr, K., and Cagan, R. (2013). A Drosophila Model of high sugar diet-induced cardiomyopathy. PLoS Genet. *9*, e1003175.

Obata, F., Tsuda-Sakurai, K., Yamazaki, T., Nishio, R., Nishimura, K., Kimura, M., Funakoshi, M., and Miura, M. (2018). Nutritional control of stem cell division through S-Adenosylmethionine in drosophila intestine. Dev. Cell *44*, 741–751.e3.

O'Brien, L.E., Soliman, S.S., Li, X., and Bilder, D. (2011). Altered modes of stem cell division drive adaptive intestinal growth. Cell *147*, 603–614.

Oikari, S., Makkonen, K., Deen, A.J., Tyni, I., Kärnä, R., Tammi, R.H., and Tammi, M.I. (2016). Hexosamine biosynthesis in keratinocytes: roles of GFAT and GNPDA enzymes in the maintenance of UDP-GlcNAc content and hyaluronan synthesis. Glycobiology *26*, 710–722.

Owen, O.E., Kalhan, S.C., and Hanson, R.W. (2002). The key role of anaplerosis and cataplerosis for citric acid cycle function. J. Biol. Chem. *277*, 30409–30412.

Port, F., Chen, H.M., Lee, T., and Bullock, S.L. (2014). Optimized CRISPR/Cas tools for efficient germline and somatic genome engineering in Drosophila. Proc. Natl. Acad. Sci. USA *111*, E2967–E2976.

Quan, Z., Sun, P., Lin, G., and Xi, R. (2013). TSC1/2 regulates intestinal stem cell maintenance and lineage differentiation through Rheb-TORC1-S6K but independently of nutritional status or Notch regulation. J. Cell Sci. *126*, 3884–3892.

Rodríguez-Colman, M.J., Schewe, M., Meerlo, M., Stigter, E., Gerrits, J., Pras-Raves, M., Sacchetti, A., Hornsveld, M., Oost, K.C., Snippert, H.J., et al. (2017). Interplay between metabolic identities in the intestinal crypt supports stem cell function. Nature *543*, 424–427.

Saxton, R.A., and Sabatini, D.M. (2017). MTOR signaling in growth, metabolism, and disease. Cell *168*, 960–976.

Schell, J.C., Wisidagama, D.R., Bensard, C., Zhao, H., Wei, P., Tanner, J., Flores, A., Mohlman, J., Sorensen, L.K., Earl, C.S., et al. (2017). Control of intestinal stem cell function and proliferation by mitochondrial pyruvate metabolism. Nat. Cell Biol. *19*, 1027–1036.

Shaw, D. (2012). Intestinal mucosal atrophy and adaptation. World J. Gastroenterol. *18*, 6357–6375.

Song, W., Veenstra, J.A., and Perrimon, N. (2014). Control of lipid metabolism by tachykinin in drosophila. Cell Rep. *9*, 40–47.

Song, W., Cheng, D., Hong, S., Sappe, B., Hu, Y., Wei, N., Zhu, C., O'Connor, M.B., Pissios, P., and Perrimon, N. (2017). Midgut-derived activin regulates glucagon-like action in the fat body and glycemic control. Cell Metab. *25*, 386–399.

Teesalu, M., Rovenko, B.M., and Hietakangas, V. (2017). Salt-inducible kinase 3 provides sugar tolerance by regulating NADPH/NADP$^+$ redox balance. Curr. Biol. *27*, 458–464.

Teleman, A.A. (2009). Molecular mechanisms of metabolic regulation by insulin in Drosophila. Biochem. J. *425*, 13–26.

Tennessen, J.M., Barry, W.E., Cox, J., and Thummel, C.S. (2014). Methods for studying metabolism in Drosophila. Methods *68*, 105–115.

Traxinger, R.R., and Marshall, S. (1991). Coordinated regulation of glutamine: fructose-6-phosphate amidotransferase activity by insulin, glucose, and glutamine. Role of hexosamine biosynthesis in enzyme regulation. J. Biol. Chem. *266*, 10148–10154.

van der Hulst, R.R., van Kreel, B.K., von Meyenfeldt, M.F., Brummer, R.J., Arends, J.W., Deutz, N.E., and Soeters, P.B. (1993). Glutamine and the preservation of gut integrity. Lancet *341*, 1363–1365.

Weimer, S., Priebs, J., Kuhlow, D., Groth, M., Priebe, S., Mansfeld, J., Merry, T.L., Dubuis, S., Laube, B., Pfeiffer, A.F., et al. (2014). D-glucosamine supplementation extends life span of nematodes and of ageing mice. Nat. Commun. *5*, 3563.

Wellen, K.E., Lu, C., Mancuso, A., Lemons, J.M.S., Ryczko, M., Dennis, J.W., Rabinowitz, J.D., Coller, H.A., and Thompson, C.B. (2010). The hexosamine biosynthetic pathway couples growth factor-induced glutamine uptake to glucose metabolism. Genes Dev. *24*, 2784–2799.

Yang, X., Ongusaha, P.P., Miles, P.D., Havstad, J.C., Zhang, F., So, W.V., Kudlow, J.E., Michell, R.H., Olefsky, J.M., Field, S.J., et al. (2008). Phosphoinositide signalling links O-GlcNAc transferase to insulin resistance. Nature *451*, 964–969.

Yeo, S.L., Lloyd, A., Kozak, K., Dinh, A., Dick, T., Yang, X., Sakonju, S., and Chia, W. (1995). On the functional overlap between two Drosophila POU homeo domain genes and the cell fate specification of a CNS neural precursor. Genes Dev. *9*, 1223–1236.

Yilmaz, Ö.H., Katajisto, P., Lamming, D.W., Gültekin, Y., Bauer-Rowe, K.E., Sengupta, S., Birsoy, K., Dursun, A., Yilmaz, V.O., Selig, M., et al. (2012). mTORC1 in the Paneth cell niche couples intestinal stem-cell function to calorie intake. Nature *486*, 490–495.

Zeng, X., Chauhan, C., and Hou, S.X. (2010). Characterization of midgut stem cell- and enteroblast-specific Gal4 lines in drosophila. Genesis *48*, 607–611.

STAR★METHODS

KEY RESOURCES TABLE

REAGENT or RESOURCE	SOURCE	IDENTIFIER
Antibodies		
Mouse monoclonal anti Delta	DSHB	C594.9B-s; RRID: AB_528194
Mouse monoclonal anti Prospero	DSHB	MR1A-c; RRID: AB_528440
Rabbit polyclonal anti phospho histone H3 Ser10	Cell Signaling	9701; RRID: AB_331535
Rabbit polyclonal anti Pdm1	Yeo et al., 1995	N/A
Mouse monoclonal anti beta-galactosidase	Promega	Z378A; RRID: AB_2313752
Chemicals, Peptides, and Recombinant Proteins		
erioglaucine disodium salt	Sigma	861146
N-acetyl-D-glucosamine	MP Biomedicals	100068
Deposited Data		
RNAseq data	GEO	GEO: GSE107052
Experimental Models: Organisms/Strains		
Drosophila melanogaster larvae, Age 1-6d after egg laying, Sex: male & female	N/A	N/A
Drosophila melanogaster adults, Age 14d, Sex: female	N/A	N/A
Drosophila melanogaster mutant Gfat2$^{\Delta 1}$	This study	N/A
Drosophila melanogaster mutant Gfat2$^{\Delta 2}$	This study	N/A
Drosophila melanogaster UAS-Gfat2	This study	N/A
Drosophila melanogaster esg-Gal4^{ts}	Jiang and Edgar, 2009	N/A
Drosophila melanogaster Su(H)GBE-Gal4^{ts}	Zeng et al., 2010	N/A
Drosophila melanogaster UAS-mCD8::GFP, hsFLP; tub- GAL4; FRT82B tub-GAL80	A gift from B. Edgar (Heidelberg Univ./Univ. of Utah)	N/A
Drosophila melanogaster UAS-InR^{DN}	Bloomington stock center	8252
Drosophila melanogaster UAS-InR^{A1325D}	Bloomington stock center	8263
Drosophila melanogaster UAS-InR$^{\alpha del}$	Bloomington stock center	8248
Drosophila melanogaster UAS-rl^{sem}	Bloomington stock center	59006
Drosophila melanogaster UAS-Akt^{myr}	Bloomington stock center	50758
Drosophila melanogaster UAS-LDH^{RNAi}	VDRC	110190
Drosophila melanogaster UAS-PDHα^{RNAi}	VDRC	107209
Drosophila melanogaster UAS-PDHβ^{RNAi}	VDRC	104022
Oligonucleotides		
Guide RNA for CRISPR mediated mutagenesis of Gfat2: AAACTACTTGACGCCCAAGT	This study	N/A
Software and Algorithms		
R/Bioconductor	N/A	https://www.bioconductor.org/
ImageJ	N/A	https://imagej.nih.gov/ij/

CONTACT FOR REAGENT AND RESOURCE SHARING

Further information and requests for resources and reagents should be directed to and will be fulfilled by the Lead Contact, Michael Boutros (m.boutros@dkfz-heidelberg.de).

EXPERIMENTAL MODEL AND SUBJECT DETAILS

Drosophila Stocks

Drosophila stocks used in this study: Gfat2$^{\Delta 1}$ and Gfat2$^{\Delta 2}$ (this study), UAS-Gfat2 (this study), Esg-Gal4^{ts} (Jiang and Edgar, 2009), Su(H)GBE-Gal4^{ts} (Zeng et al., 2010), UAS-mCD8::GFP, hsFLP; tub-GAL4; FRT82B tub-GAL80 (a gift from B. Edgar), UAS-InR^{DN}

(BLN:8252), UAS-InR^{A1325D} (BLN:8263), UAS-InR$^{\alpha del}$ (BLN:8248), UAS-rl^{sem} (Erk^{CA}, BLN:59006), UAS-Akt^{myr} (BLN:50758), UAS-LDH^{RNAi} (VDRC:110190), UAS-PDHα^{RNAi} (VDRC:107209), UAS-PDHβ^{RNAi} (VDRC:104022). Fly stocks were maintained at 25°C with fly food containing agar 0.8% (w/v), syrup 4,4% (w/v), corn flour 8% (w/v), soya flour 1% (w/v), malt 8% (w/v), dry baker's yeast 1.8% (w/v), propionic acid 0.6% (v/v), phosphoric acid 0.06% (v/v) and Nipagin (methylparaben) 0.24% (v/v). For calorie restriction experiments, the fly food was diluted to 0.25x in 0.8% agar while keeping the preservative concentrations constant. For GlcNAc feeding, N-acetyl-D-glucosamine (MP Biomedicals, cat no:100068) was directly dissolved into the fly food in 0.1 M concentration.

METHOD DETAILS

MARCM Analysis

Fly stocks were crossed to UAS-mCD8::GFP, hsFLP; tub-GAL4; FRT82B tub-GAL80 to generate offspring with the desired genotype. Newly eclosed mated female flies were kept in standard fly food for seven days before clone induction. To induce clones, flies were transferred into indicated diets and heat-shocked at 37°C for 1 hour 30 minutes in a water bath. Intestines were dissected and analyzed seven days after the heat shock. To count cell number in clones, low-resolution confocal Z-stacks were taken from midgut R4c region (Buchon et al., 2013). The stacks were processed by the ImageJ software, and the cells within clones were scored by superimposing GFP and DAPI channels.

Food Intake Measurement

Female flies of the genotype UAS-mCD8::GFP, hsFLP; tub-GAL4/+; FRT82B tub-GAL80/FRT82B were sampled in parallel with MARCM clone induction. Before measuring food intake the flies were kept on the indicated fly food for 5 days. The flies were then transferred to the indicated diet supplemented with 0.5% (w/v) Acid Blue 9 (erioglaucine disodium salt, Sigma 861146) for 4 hours. Quadruplicates of 8 flies per sample were then homogenized in 500μl PBS and cellular depris was removed by centrifugation. Food intake was quantified by measuring absorbance of the supernatant at 625nm and normalized to the wet weight of the flies.

Generation of Drosophila Mutants

The *gfat2*$^{\Delta 1}$ and *gfat2*$^{\Delta 2}$ alleles were generated by CRISPR/Cas9 as described previously (Port et al., 2014). The gRNA sequence used was AAACTACTTGACGCCCAAGT. Deletions were confirmed by Sanger sequencing.

Pupation Curves and Lethality Measurement

Thirty first-instar larvae were seeded per vial, four vials for each genotype, and grown under controlled conditions, in the indicated diets as described above. The number of pupated animals was counted over time, and is represented as a percentage of total pupated animals. For measuring larval lethality, thirty first-instar larvae were seeded per vial, four vials per genotype, and grown on 5% sucrose in 0.5% agar supplemented with 0.1 M GlcNAc. The number of surviving animals was counted over time, and is represented as a percentage of total animals.

Immunohistochemistry

For immunofluorescence staining, intestines were dissected in PBS and fixed in 8% paraformaldehyde for 30 min. Tissue were washed with 0.1% Triton-X100 in PBS, and blocked in 1% bovine serum albumin for 1 h. Subsequently, tissues were stained with anti-Delta (1:120) (C594.9B, Developmental Studies Hybridoma Bank, DSHB), anti-Prospero (MR1A-c, DSHB) anti-Pdm1 (1:2000) (Yeo et al., 1995, a gift from W. Chia) and anti-pH3 (1:600) (Cell Signaling, cat no: 9701). The samples were mounted in Vectashield and imaged by the Broadband Confocal Leica TCS SP5 and SP8 systems.

RNA-Seq and Data Analysis

For RNA sequencing, 7-day-old mated females were placed to calorie restriction (0.25x diluted food) with 0.1 M GlcNAc for 24 hours. Subsequently, intestines were dissected and total RNA was extracted using the RNeasy kit (Qiagen). Four independent samples from control (-GlcNAc) and experiment (+GlcNAc) were sampled in parallel. The samples were sequenced on Illumina HiSeq 2500 platform (single-end reads, length 50 bp).

The quality of the raw sequencing data was assessed with FASTQC (v.0.11.2) and reads were trimmed with Trimmomatic (v.0.33). The reads were required to be minimum of 36 bases long, and they were scanned with 4-base sliding window with minimum quality value of 15 per base. The strands were also required minimum quality of 20 in both ends. TopHat (v.2.1.0) was used for mapping reads to the *D. melanogaster* reference genome (Flybase R6.10). The HTSeq was used for strand-specific quantification of exons with reads below quality of 10 discarded. The differential expression analysis was performed with R/Bioconductor package limma. Low expressed genes were filtered, expressed genes requiring to have cpm>1 in at least 3/4 replicates in at least one of the conditions. For the gene set enrichment analysis we used R/Bioconductor package piano (v.1.1.16.2). Command runGSA was used with GSEA algorithm and row sampling with 1000 permutations for all expressed genes. The pathway databases consisted of KEGG, Reactome, Wikipathways and GO. The heatmaps of selected pathways were performed using scaled $\log_2$ CPM values of each replicate. The row-wise clustering of the heatmaps was performed using correlation distance.

DATA AND SOFTWARE AVAILABILITY

The accession number for the RNAseq data reported in this paper is GEO: GSE107052.

QUANTIFICATION AND STATISTICAL ANALYSIS

Statistical analyses were performed in R/Bioconductor. For the count data Wilcoxon rank-sum test with multiple testing correction (FDR<0.05) was used. For the parametric data two-way ANOVA in conjunction with Tukey's HSD test was used. The number of samples (N^{guts} & N^{clones}) is detailed within figures and in Table S3.

Developmental Cell, Volume *47*

Supplemental Information

Stem Cell Intrinsic Hexosamine Metabolism Regulates Intestinal Adaptation to Nutrient Content

Jaakko Mattila, Krista Kokki, Ville Hietakangas, and Michael Boutros

FIGURE S1 related to figure 2

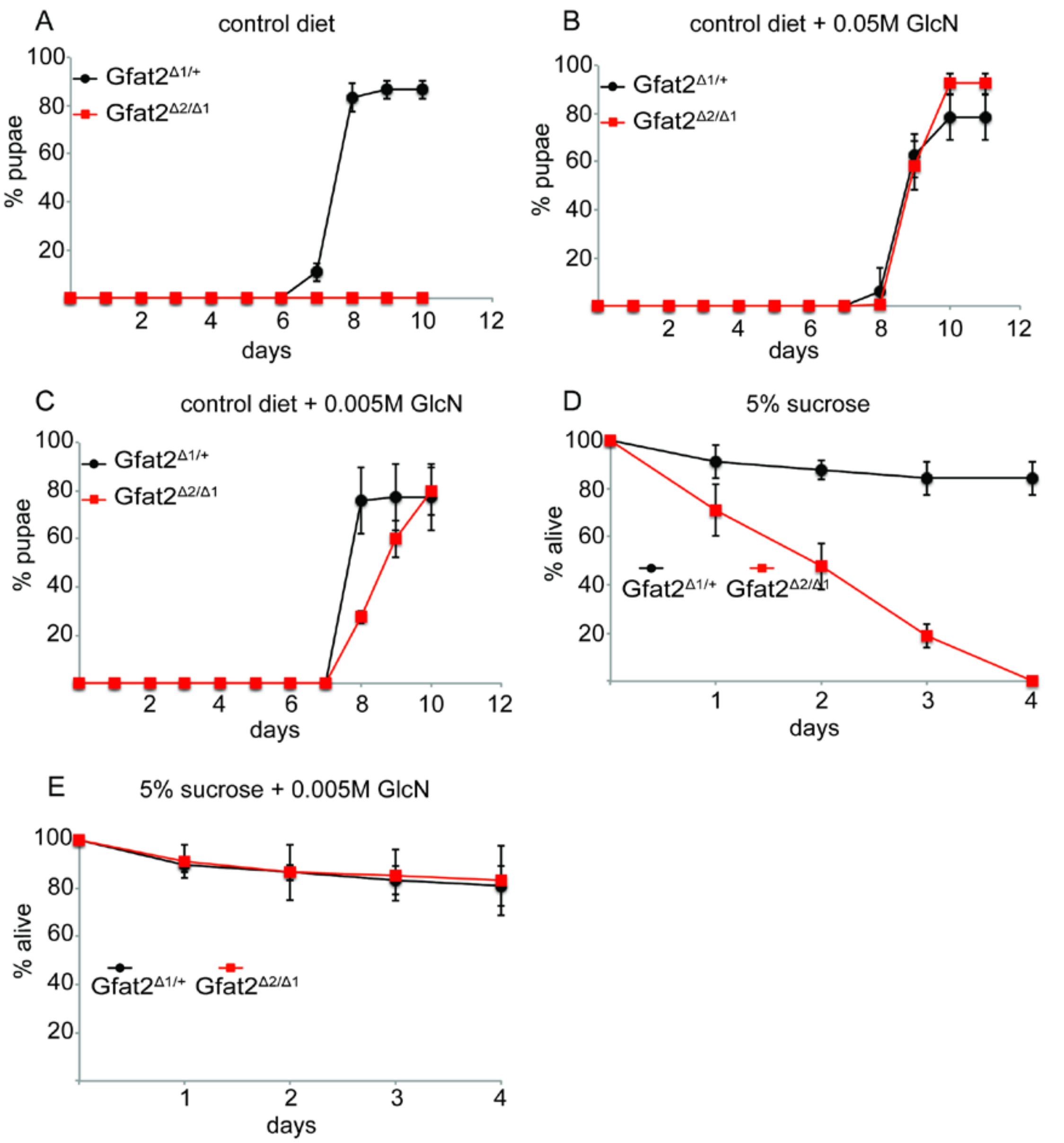

Figure S1. *gfat2* mutant lethality is rescued by GlcNAc feeding. Related to Figure 2.

(A-C) Pupation curves of *gfat2*$^{\Delta 1/\Delta 2}$ trans-heterozygote and *gfat2*$^{\Delta 1/+}$ controls fed in control (A) and GlcNAc supplemented diets (B & C). (D-E) Lethality of *gfat2*$^{\Delta 1/\Delta 2}$ trans-heterozygote and *gfat2*$^{\Delta 1/+}$ control first instar larvae in 5% sucrose (D) and 5% sucrose supplemented with GlcNAc (E). Error bars are standard deviation of the mean from three replicate experiments conducted in parallel.

FIGURE S2 related to figure 2

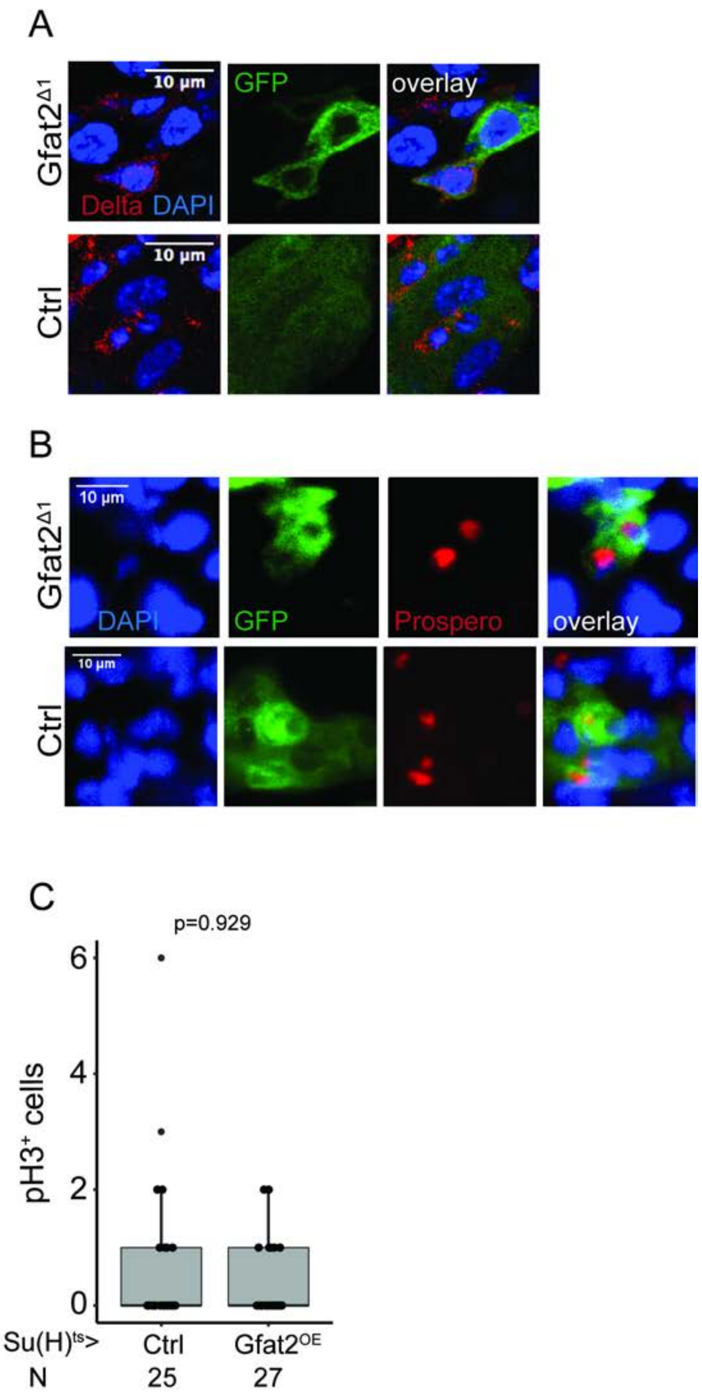

Figure S2. ISCs of the *gfat2$^{\Delta 1}$* intestinal clones are viable and able to undergo asymmetric cell divisions and EE lineage differentiation. Related to Figure 2.

(A) *gfat2*$^{\Delta 1}$ and control MARCM clones stained with the ISC marker anti-Delta antibody 14 days after clone induction. (B) *gfat2*$^{\Delta 1}$ and control MARCM clones stained with the EE marker anti-Prospero 7 days after clone induction. (C) Overexpression of Gfat2 by the Su(H)-Gal4^{ts} driver does not change midgut mitotic index. Quantification of the pH3 positive cells from Su(H)-Gal4^{ts}>control and Esg-Gal4^{ts}>UAS-Gfat2 intestines. P-values are calculated by Wilcoxon rank-sum test.

FIGURE S3 related to figure 3

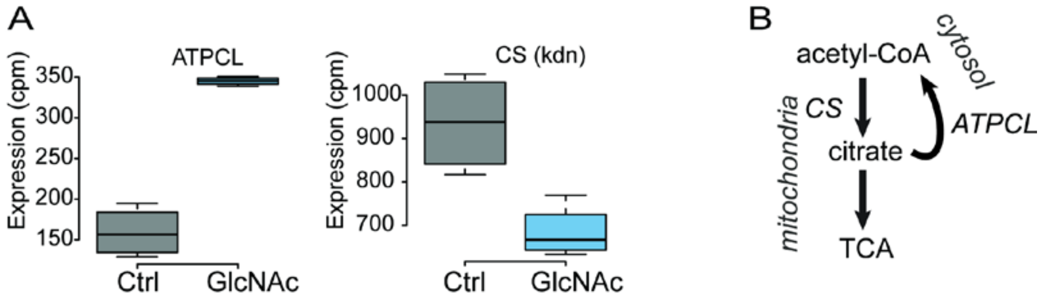

Figure S3. HBP induced transcriptional changes in genes involved in citrate metabolism. Related to Figure 3.

(A) mRNA expressions (counts per million, cpm) of ATP citrate lyase (ATPCL) and Citrate synthase (CS) (kdn) on Ctrl vs. GlcNAc diet. (B) Schematics of the role of CS and ATPCL in citrate metabolism driving the activities of TCA cycle and fatty acid biosynthesis, respectively.

FIGURE S4 related to figure 4

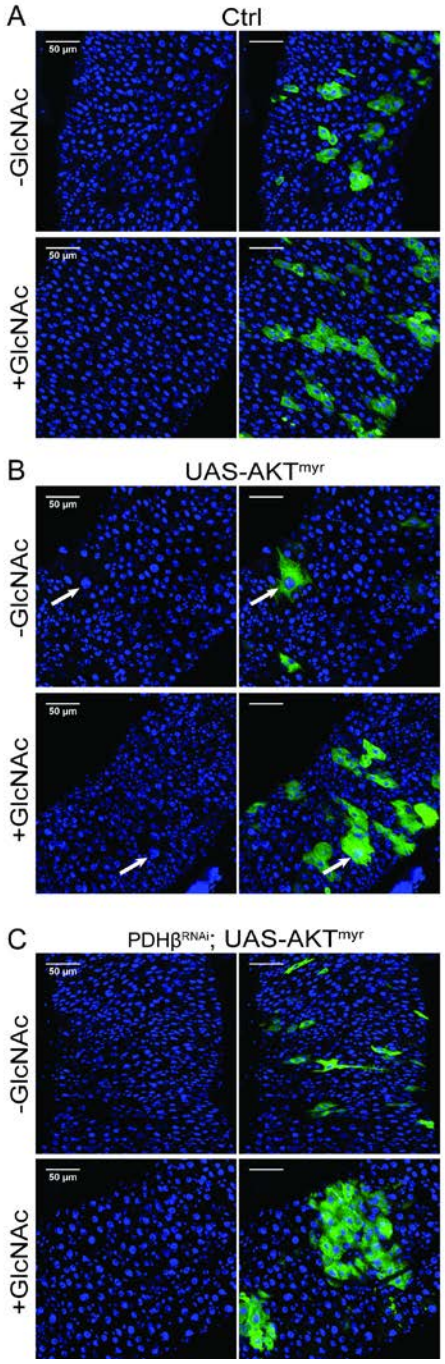

Figure S4. The regulatory output of Akt^{myr} expression in intestinal MARCM clones is determined by dietary GlcNAc and pyruvate metabolism. Related to Figure 4.

Midgut MARCM clones of control (A), UAS-Akt^{myr} (B) and PDHβ^{RNAi}; UAS-Akt^{myr} (C) with or without dietary GlcNAc. Arrows in (B) point to enlarged cells.

Table S3. Number of samples used in the clonal experiments. Related to Figures 1, 2, 3 & 4

Figure	Genotype	Diet	N guts	N clones
1D	MARCM>Ctrl	1x -GlcNAc	12	209
1D	MARCM>Ctrl	1x +GlcNAc	14	224
1D	MARCM>Ctrl	0.25x -GlcNAc	14	209
1D	MARCM>Ctrl	0.25x +GlcNAc	11	146
2F	MARCM>Ctrl	1x -GlcNAc	12	242
2F	MARCM>$Gfat2^{\Delta1}$	1x -GlcNAc	10	176
2F	MARCM>$Gfat2^{\Delta1}$	1x +GlcNAc	11	152
2F	MARCM>UAS-Gfat2; $Gfat2^{\Delta1}$	1x -GlcNAc	7	92
2G	MARCM>Ctrl	1x -GlcNAc	12	216
2G	MARCM>Ctrl	0.25x -GlcNAc	8	136
2G	MARCM>UAS-Gfat2	1x -GlcNAc	14	297
2G	MARCM>UAS-Gfat2	0.25x -GlcNAc	14	252
3F	MARCM>Ctrl	0.25x -GlcNAc	10	217
3F	MARCM>Ctrl	0.25x +GlcNAc	9	136
3F	MARCM>PDHα RNAi	0.25x -GlcNAc	11	187
3F	MARCM>PDHα RNAi	0.25x +GlcNAc	11	177
3F	MARCM>PDHβ RNAi	0.25x -GlcNAc	9	177
3F	MARCM>PDHβ RNAi	0.25x +GlcNAc	9	141
3F	MARCM>LDH RNAi	0.25x -GlcNAc	8	146
3F	MARCM>LDH RNAi	0.25x +GlcNAc	10	169
4A	MARCM>Ctrl	0.25x -GlcNAc	12	210
4A	MARCM>Ctrl	1x -GlcNAc	14	214
4A	MARCM>$Gfat2^{\Delta1}$	0.25x -GlcNAc	13	224
4A	MARCM>$Gfat2^{\Delta1}$	1x -GlcNAc	13	251
4D	MARCM>Ctrl	1x -GlcNAc	10	139
4D	MARCM>Ctrl	1x +GlcNAc	10	141
4D	MARCM>UAS-InR^{DN}	1x -GlcNAc	9	184
4D	MARCM>UAS-InR^{DN}	1x +GlcNAc	10	110
4D	MARCM>UAS-InR^{A1325D}	1x -GlcNAc	11	114
4D	MARCM>UAS-InR^{A1325D}	1x +GlcNAc	13	177
4D	MARCM>UAS-$InR^{\alpha del}$	1x -GlcNAc	12	231
4D	MARCM>UAS-$InR^{\alpha del}$	1x +GlcNAc	9	140
4D	MARCM>UAS-Erk^{CA}	1x -GlcNAc	11	152
4D	MARCM>UAS-Erk^{CA}	1x +GlcNAc	13	176
4F	MARCM>Ctrl	1x -GlcNAc	10	248
4F	MARCM>UAS-Akt^{myr}	1x -GlcNAc	10	69
4F	MARCM>UAS-Gfat2; UAS-Akt^{myr}	1x -GlcNAc	10	263
